# Effects of multiple modes of UltraPulse fractional CO_2_ laser treatment on extensive scarring: a retrospective study

**DOI:** 10.1007/s10103-021-03406-x

**Published:** 2021-08-26

**Authors:** Xiaojing Ge, Yute Sun, Jing Lin, Fang Zhou, Gang Yao, Xin Su

**Affiliations:** Department of Plastic and Burn Surgery, The First Affiliated Hospital of Nanjing Medical University, 300 Guangzhou Rd, Nanjing, 210029 Jiangsu Province China

**Keywords:** Scars, UltraPulse fractional CO_2_ laser, Laser therapy, Supramaximal-area scarring

## Abstract

The main therapeutic options for extensive scarring (e.g., > 20% of the total body surface area, or TBSA) after burns and trauma have focused on conservative treatments, such as compression, moisturization, and topical agent application. However, these treatments may not achieve optimal effects due to the large size and complexity of the scars. UltraPulse fractional CO_2_ laser treatment is a novel approach that is currently a subject of intense interest; this treatment is most widely used to improve texture, pliability, and pigmentation in all types of scars. However, no studies on the independent use of UltraPulse fractional CO_2_ laser treatment for extensive scars have been reported. This retrospective study evaluated a total of 21 patients, whose scars covered 20 to 65% TBSA. Scar thickness was measured by ultrasonography before treatment. Personalized treatment modalities and parameters were set according to the scar type and thickness. Scar formation and treatment effects were evaluated by photography, the Patient and Observer Scar Assessment Scale (POSAS), and patients’ judgment of effectiveness. Where the scars covered joints, joint function was assessed by measuring the maximum range of motion (ROM). With laser therapy, scars became flatter and lighter; furthermore, pruritus, pain, and discomfort decreased significantly. POSAS scores significantly decreased after laser therapy, including the item scores for pain and pruritus. There were no instances of joint contracture, ROM reduction, apparent functional impairment, serious adverse events, or comorbidities. This study demonstrates the safety and efficiency of UltraPulse fractional CO_2_ laser treatment for extensive scarring.

**Supplementary Information:**

The online version contains supplementary material available at 10.1007/s10103-021-03406-x.

## Introduction

Extensive trauma and severe burns (ETSB) bring great suffering to patients, impair their quality of life, and affect cosmetic appearance and functionality [1]. In the past, scarring was often overlooked in the early stages of wound healing until functional impairment was present. Due to further progress, more doctors and patients have become aware of the need to consider scarring from the beginning of wound healing, and further attention is being paid to mobility, appearance, and relief of pain and itching early in the healing process [2, 3]. We consider scarring to be severe when it meets the following two conditions: (1) there is a nonlinear, patchy distribution of scar tissue covering a large enough area that it is difficult to conceal the site with regular clothing and (2) the scar covers at least one joint. In our experience, these conditions tend to be met when the area of scarring is > 20% of the total body surface area (TBSA); therefore, we defined 20% TBSA as the upper threshold of non-severe scarring, and we defined anything above this threshold as supramaximal-area scars (SASs). Patients with SASs were selected for this study. An SAS may include multiple scar types simultaneously and change dynamically over time (Sup. 1). Hypertrophic scars (HSs) can cause itching, pain, and discomfort; scar hyperplasia/contracture can cause joint dysfunction. With scarring over an extensive area, the treatment needs become more complex.

Traditional burn scar management methods include compression, moisturization, massage, topical drugs, local injections of glucocorticoids, radiofrequency, radionuclides, cryotherapy, and surgery [4–6]. Since so many parts are involved in SASs, these regimens are usually unsuitable, and even partial scar treatment poses challenging multifaceted problems that make it difficult to achieve satisfactory results, such as unbearable itchy discomfort. For SAS patients, there are limited donor sites for surgical repair [7]. Thus, the ideal treatment for SASs requires as few different methods as possible, can be performed in the early stages, uses convenient treatment modalities, shows clear effectiveness, and does not damage normal tissues.

Evidence suggests that laser treatment is an effective therapeutic modality for all types of scars. In HSs 1 year after burn injury, significant and sustained improvements in the elasticity, thickness, appearance, and symptoms of the scars were observed after 1–3 sessions with complex ablative CO_2_ laser treatment alone [8, 9]. Laser treatments for scar tissue have been increasingly recognized by academics and a variety of guidelines [10–13], but further evaluation is still needed to determine the effectiveness and safety of laser treatment for SASs. Given the diversity and disparity of SASs, we applied various complex modes of UltraPulse fractional CO_2_ laser treatment to different scars in a personalized and holistic unified approach. This study is the first to evaluate the safety, feasibility, and effectiveness of fractional CO_2_ laser treatment for SASs over a long-term follow-up period.

## Materials and methods

This retrospective study enrolled patients with SASs after ETSB from September 2017 to November 2019, and the ethics committee had approved this study.

### Inclusion criteria

1. Age 15–60 years
2. SASs after ETSB and 4 weeks to 12 months of wound healing
3. Scars covering ≥ 20% of the TBSA
4. Complete case data with follow-up

### Exclusion criteria

1. Joint ROM reduction of at least 50% of the normal range or organ displacement requiring surgery
2. Foci of infection near the areas to be treated
3. Pregnancy or nursing at the time of the study; severe cardiovascular disease or organ failure
4. Use of chemotherapy or systemic hormone and immunosuppressive therapy in the previous 6 months
5. Incomplete case data

Effectiveness was assessed by comparing and analyzing measurements from before and after treatment. The treatment outcomes of interest were evaluated using digital photographic documentation, Patient and Observer Scar Assessment Scale (POSAS) scores, and maximum joint ROM.

### Laser treatment

The equipment involved in this study was an UltraPulse fractional carbon dioxide (CO_2_) laser (Lumenis Ltd., Yokneam, Israel) with a wavelength of 10,600 nm, energy of 20 ~ 175 mJ, power of 1 ~ 60 W, a frequency of 30 ~ 300 Hz, and a density of 3 ~ 5%.

The scar thickness was measured using ultrasonography at a few apparent high-tension spots on each SAS. The appropriate handpiece and energy level were chosen according to the thickness of the scar (Table 1). Hypertrophic scars (> 4 mm) and stretched scars were treated in the two‐passes protocol, and others were treated in the one pass.

**Table 1:** Scar types and treatment modalities

	Thickness	UltraPulse mode	Active FX mode	SCAAR FX mode	Deep FX mode
Hypertrophic scars	≤ 1.5 mm		√		
Hypertrophic scars	1.5–4.0 mm			√	
*Hypertrophic scars	> 4 mm	√		√	
*Stretched scars	Contracture	√		√	
Superficial or erythematous scars			√		
Uneven area					√
Residual trauma					√

^*^For scars that were > 4 mm thick or whose contracture interfered with the functionality of underlying joints, patients were treated with combination therapy. In the first pass, which focused on depth, puncta were treated with UltraPulse mode to reach the deep part of the scar tissue. The puncta were spaced 4–5 mm apart to avoid excessive heat damage caused by overlapping thermal effects. The second step of the combination therapy focused on breadth; in this pass, SCAAR FX mode was used for uniform scanning

### Treatment protocol

General or local anesthesia (infiltration or nerve block anesthesia) was selected depending on the patient’s preference and age. Surface anesthesia for approximately 20–30 min with compound lidocaine cream (1 g of cream including 25 mg of prilocaine and 25 mg of lidocaine) (Beijing Ziguang Pharmaceutical Co., Ltd., Beijing, China) or local anesthesia such as brachial plexus block anesthesia and iliac fascial space block anesthesia (0.2% ropivacaine, 30 mL) was performed by an anesthesiologist. At the beginning of the procedure, the site was sterilized three times with 75% alcohol. After treatment, moist exposed burn ointment (Shantou MEBO Pharmaceutical Co., Ltd., China) was smeared across all the ablative laser microcolumns and reapplied every 4–6 h. The epidermal basement membrane was completely re-epithelized in approximately 10–14 days. Each treatment period was separated from the next by an interval of 12 weeks. Each patient was followed up at least 6 months after the end of the session. Records were made of the healing time after laser treatment and any adverse reactions such as erythema or infection.

### Assessment of treatment effectiveness

Photographs were taken before each therapy session and 4 weeks after the final treatment; two clinicians who were blinded to the treatments took the photos in a consistent environment. Each clinician completed the POSAS; additionally, each patient was asked to judge the effectiveness of treatment, and the clinicians measured the maximum ROM of each effected joint. The primary index was the POSAS score, which reflects vascularization, pigmentation, thickness, relief, pliability, and patients’ self-perception.

The secondary indicators included the patients’ judgment of effectiveness and the maximum joint ROM. Therapeutic effectiveness judgments were analyzed as follows. If a patient judged that the scars improved by > 50% overall, the outcome was classified as significant. If the patient-reported improvement was 25–50%, the treatment was considered effective; if the patient reported dissatisfaction, increased scarring, or < 25% improvement, the treatment was considered ineffective. The overall effectiveness rate was calculated as follows: overall effectiveness = (significant + effective)/total cases × 100%. The ROM of a joint was considered restricted if it was less than 50% of its normal range purely as a result of scar contracture. X‐rays were obtained to rule out skeletal problems.

### Statistical methods

Categorical variables are expressed as counts and percentages. The independent *t*-test was used for numerical data. Descriptive data are presented as the mean and standard deviation for quantitative variables or the frequency for categorical variables. Statistical significance was defined as *P* < 0.05. SPSS 26.0 for Windows was used for all statistical analyses.

## Results

A total of 21 patients with SASs after ETSB were enrolled; their scars covered 20–65% TBSA, with an average of 29% TBSA. The average sessions were (4.86 ± 1.74). The shortest interval between wound healing and laser treatment was 4 weeks, and the longest was 12 months. Sixteen patients (76%) received laser treatment within 6 months, and the other 5 patients (24%) were treated more than 6 months after the wound healed; the average interval was 5.5 months. There were 11 patients (52%) with burn scars and 10 patients (48%) with posttraumatic scars. The scars were located on the face, extremities, anterior chest, and/or the perineum; in 7 cases (58%), there were scars at multiple sites. All cases involved scars across joints; a total of 25 joints were affected, including the knee, ankle, wrist, and elbow. Three patients had scattered residual trauma in the scarred areas other than scar-related wounds; these injuries were significantly diminished in size after laser treatment. Two of them, both measuring < 2.25 cm^2^ in area, healed in 9 and 10 days (Table 2).

**Table 2:** Patient demographics

Characteristic	No
Sex	
Female	13
Male	8
Age	
Mean	31.4
Range	15–47
Cause of initial injury	
Trauma	10
Burn	11
Scar area (TBSA)	
Mean	29%
Range	20–65%
No. and type of joints	
Knee	11
Ankle	3
Wrist	3
Elbow	4
Average time between initial injury and first laser intervention	5.5 months
Range	4–12 months

There were no adverse reactions or complications, and all patients completed the laser treatment process with normal wound healing of the ablative laser microcolumns. There was no significant difference in the healing time of the laser-treated sites according to the modality of treatment (*P* > 0.05).

### Primary outcomes—POSAS score analysis

**Fig. 1:**
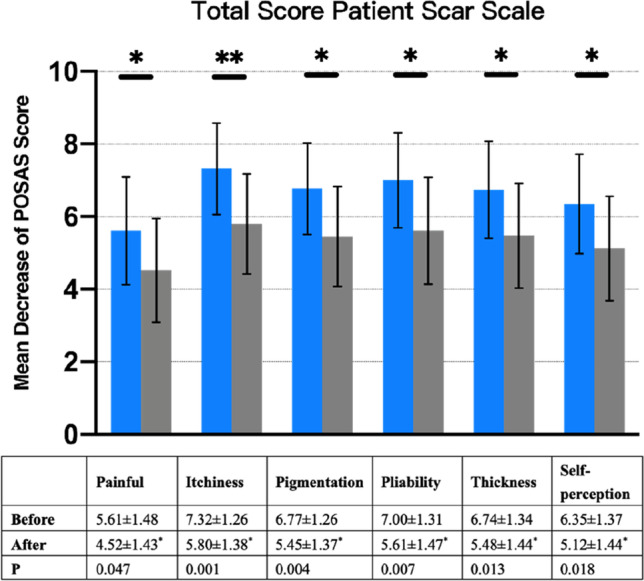
Total patient scar score

**Fig. 2:**
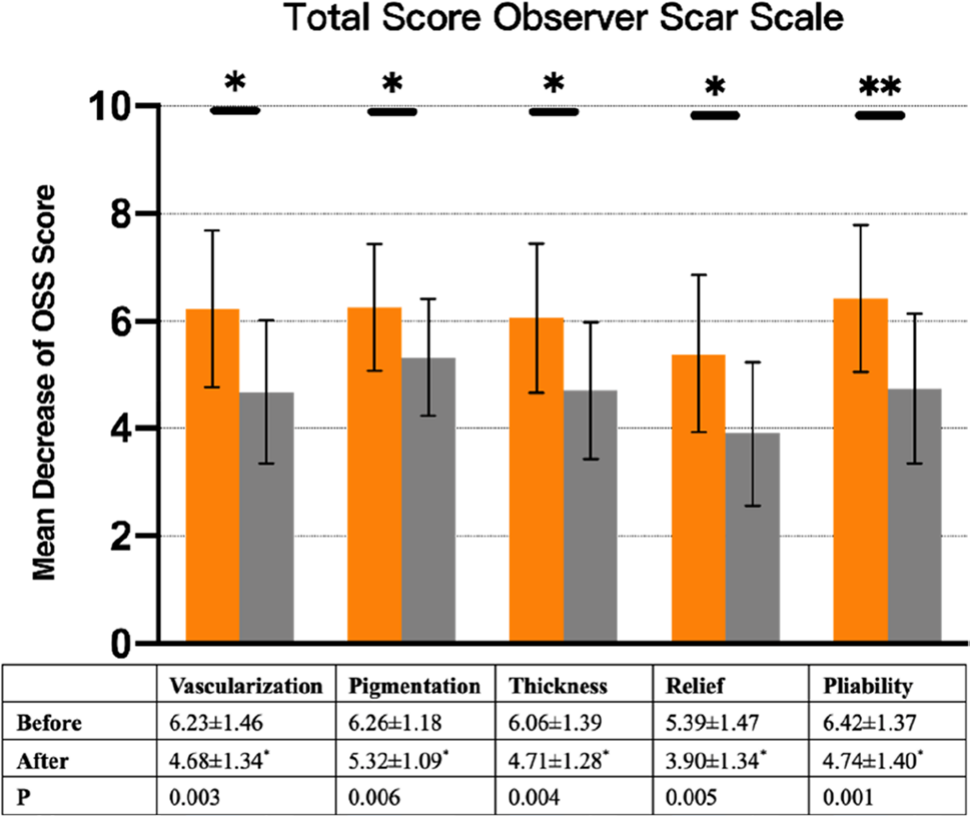
Total observer scar score

From the pretreatment baseline to the 3-month posttreatment follow-up, all patients had a significant decrease in POSAS scores (70.03 ± 17.49 before treatment vs. 55.03 ± 18.19 after treatment; *P* = 0.002). Both the patient and observer assessment scores were significantly decreased from baseline to follow-up. There was a significant improvement in scar pruritus (7.32 ± 1.58 vs. 5.80 ± 1.90; *P* = 0.001). All the remaining items were significantly changed as well (Figs. 1 and 2).

### Secondary outcomes—therapeutic effectiveness judgments and maximum ROM

All patients were satisfied with the treatment, for an overall effectiveness rate of 100%; among them, 15 patients were considered to have very satisfactory results (significant outcome rate, 71.4%); the other 6 patients had moderate improvement (effective outcome rate, 28.5%).

**Fig. 3:**
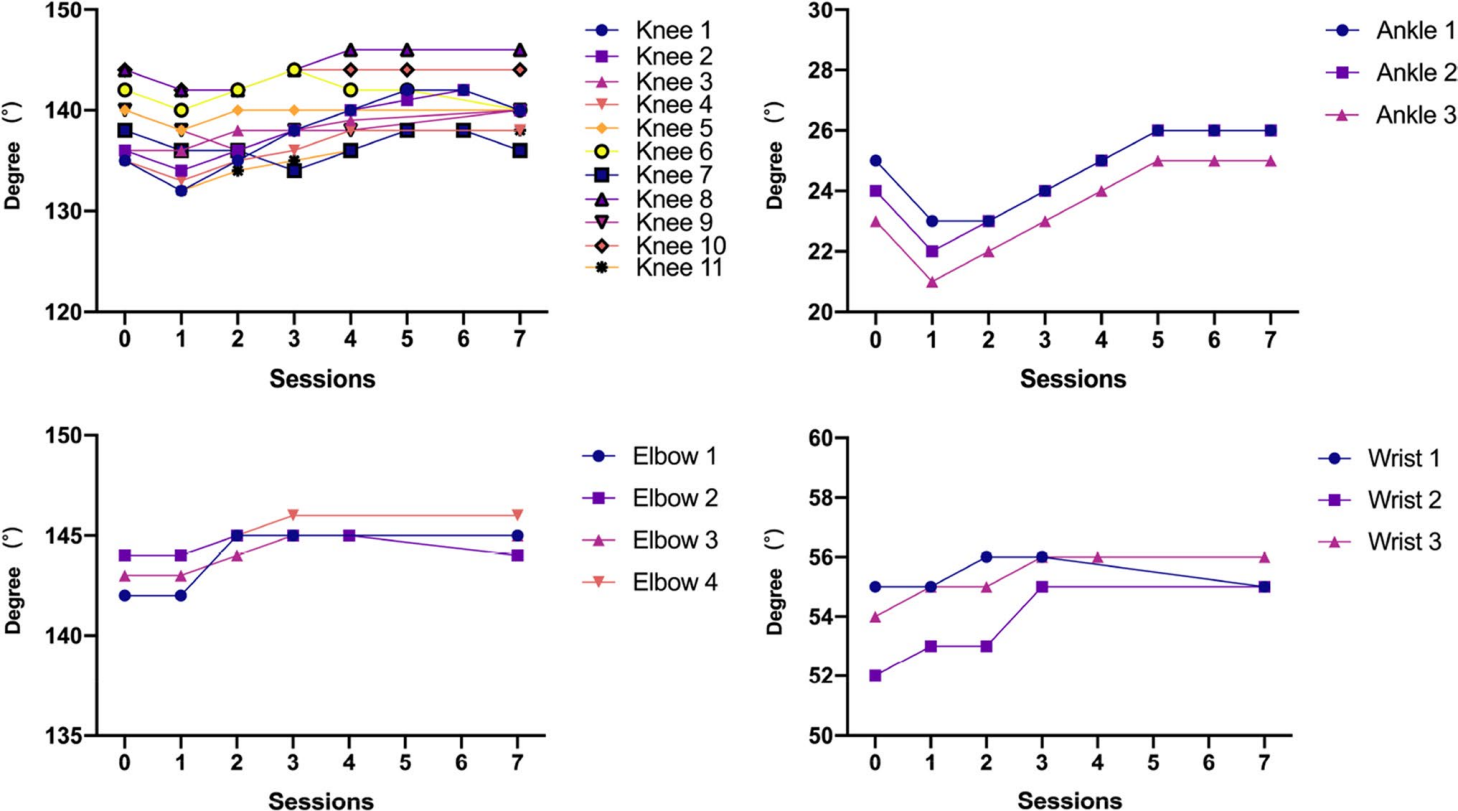
Range of motion (ROM)

All joints covered by the scars maintained a normal ROM, with no dysfunction (Fig. 3).

## Discussion

There are numerous ways to treat scars, but few articles have reported on the modalities and safety of SAS treatments. Evidence from recent years suggests that lasers are effective in treating multiple types of scars, and fractional laser treatments, especially ablative fractional laser (AFL) therapy, have the greatest potential to treat the entire range of clinical issues with a single modality [13]. Therefore, we focused on the different types of scars involved in SASs and applied personalized treatment modalities with an UltraPulse fractional CO_2_ laser.

A possible mechanism through which laser treatment improves a scar’s appearance is the alteration of its collagen structure, causing the disorganized tissue structure to be reprogrammed [14]. In the early stages of skin remodeling, laser therapy alters the type I/type III collagen ratio; regulates the expression of MMP-1, growth factors, fibroblast-specific markers, miR-18a, and miR-19a; and induces the expression of the Wnt5a, CYR61, and HSP90 genes [15, 16]. CO_2_ laser treatment, which can be performed in several modalities, is an effective treatment for most scars [8, 9, 17–19]. This may be due to the deep dermal penetration of energy that can be achieved with this device [20]. Our treatment protocols consisted of one pass for uncontracted scars < 4 mm thick and 2 passes for scars with contracture and/or a thickness of > 4 mm. The first pass of the UltraPulse laser remodeled the tissue and broke down the superficial layer of disarrayed collagen fibers within its gasification range of 4 mm, releasing the contracture around the joints. UltraPulse mode (allowing the specific, temporally concentrated release of energy) forms many microcolumns of tissue deep in the scar while creating an annular zone of coagulation surrounding each cavity [21]. Its penetration also provides a cutting action that releases contracture by severing cord-like fibers. When the first pass was complete, the second pass was performed in SCAAR mode to reach the entire depth and breadth of the scar with the thermal effect of the laser. Before treatment, ultrasonography must be performed to assess scar thickness. Our results suggest that UltraPulse mode combined with SCAAR mode is safe and effective in the treatment of scars. It has been demonstrated histologically that variable depths of dermal tissue are ablated depending on the energy of the treatment pulses [21–23].

In the early stages of scar formation, pain and pruritus bring great physical and psychological distress to patients, sometimes even including depression and anxiety. According to statistics, 87% of burn patients discharged from hospitals have pruritus symptoms [24, 25]. Mechanistically, these symptoms may be caused by the local hypoxic environment stimulating nerve endings due to congestion and hyperplasia of hypertrophic scars. Dense nerve fiber growth after tissue injury and the accompanying increase in substance P levels in nerves may also be responsible for scar pruritus [26]. The upregulation of inflammatory cytokines, such as tumor necrosis factor (TNF)-α, transforming growth factor (TGF)-β, and interleukins, may be responsible for neuropathic pain associated with burn scars [27]. The specific mechanisms by which scar pain and pruritus occur are not clear. Recent research suggests that various measures to inhibit scar growth and promote scar maturation may reduce the discomfort of pain and pruritus, including oral medications, compression therapy, silicone gel products, steroid injections, 5-fluorouracil and laser therapy, and combination therapy [28], but these treatments have not been sufficiently effective in patients with SASs after ETSB because these burns affect too many body parts. Hormones and 5-fluorouracil, although effective, cannot be used on large areas. The use of a CO_2_ laser in SASs can significantly improve patients’ subjective symptoms, such as pruritus, with none of the abovementioned drawbacks. The mechanism is still unclear, but it is speculated to involve the inhibition of new blood vessel formation and a reduction in the levels of inflammatory factors [29]. In our study, we found the same results regarding subjective symptoms: the patients’ pain and itch scores were significantly lower after CO_2_ laser treatment than before. We observed no side effects associated with this symptom relief.

Elastin decreases or even disappears at the lesion site in the 5 years following a burn, reducing the flexibility of the scar and increasing its height [1]. Hypertrophic scars are more likely to form after skin trauma in high-tension and high-stretch areas, possibly due to tension-induced fibroblast variation [30, 31]. Thus, scars across the joints may lead to dysfunction of the joints [9]. In the past, the dysfunction caused by scars often went unaddressed until the scars matured or caused severe dysfunction, at which time they were treated surgically [32]. Nevertheless, after extensive burn trauma, the body’s skin/flap supply area is limited even when dilators are used. Early prevention of dysfunction is vital, with traditional methods including compression therapy, topical medications, and rehabilitation [5]. However, dysfunction is still unavoidable due to the depth of injury, repair methods, and imperfect patient compliance. We applied UltraPulse mode to address the contracture of cord-like scar; this mode had two advantages. First, the cutting pattern of the laser was used to loosen the scar. Second, posttreatment collagen rearrangement changed the flexibility and texture of the scar, preventing further progression and joint dysfunction and conferring a therapeutic advantage. CO_2_ laser treatment is also effective on contracted scars, and the treatment effect lasts at least 6 months [33]. In our study, the earliest laser treatment took place 4 weeks after injury, and the recipient did not show joint dysfunction even after a 1-year follow-up. There is also a professional consensus that early laser treatment can delay the need for further treatment by at least a year [13].

We did not exclude the presence of remnant wounds in the early stages. Instead, we found that low-energy laser treatment of the remnants of scars resulted in faster reduction and even healing of remnant wounds. Shumaker and colleagues [34] previously reported that laser therapy promoted the development of scars related to wound healing; additionally, Tania et al. [35] found that laser therapy was effective in the treatment of chronic lower extremity ulcers in elderly patients after trauma, suggesting that lasers may stimulate healing by causing microtrauma to the wound bed, producing cytokines associated with acute injury, and destroying bacterial biofilms in the wound.

Some studies have shown up to 50% improvement in the POSAS score with natural history or burn scar maturation [36]. Evidence shows that nearly half of patients still have scarring consequences; thus, early intervention to treat scarring and prevent complications has become the mainstream direction of scar treatment. In this research, all patients achieved good treatment results and delayed or avoided joint dysfunction. However, this study is limited by its retrospective nature, inclusion of a small number of nonrandomized patients, and short follow-up period. Overall, current results support the safety of the laser treatment of SASs.

## Conclusions

The UltraPulse fractional CO_2_ laser, with multiple modes, provides safe, effective, and efficient treatment for SASs after severe burns and trauma. This treatment can be applied early in the healing process to flatten and soften both hypertrophic scars and keloids, reduce uncomfortable subjective symptoms such as itchiness and pain, and prevent joint dysfunction after treatment.

## Supplementary Information

Below is the link to the electronic supplementary material.

Supplementary file1 (DOCX 1318 KB)

## Data Availability

No data are available due to the conditions of the ethics approval.
