# Supplementary material for: Effects of multiple modes of UltraPulse fractional CO2 laser treatment on extensive scarring: a retrospective study
Source: Lasers Med Sci. 2021 Aug 26;37(3):1575–82. doi: 10.1007/s10103-021-03406-x (PMC8971167; doi:10.1007/s10103-021-03406-x)
Supplement: Supplementary file 1 — Supplementary file1 (DOCX 1318 KB) [file 10103_2021_3406_MOESM1_ESM.docx]

Sup. 1. Various coexisting types of SASs


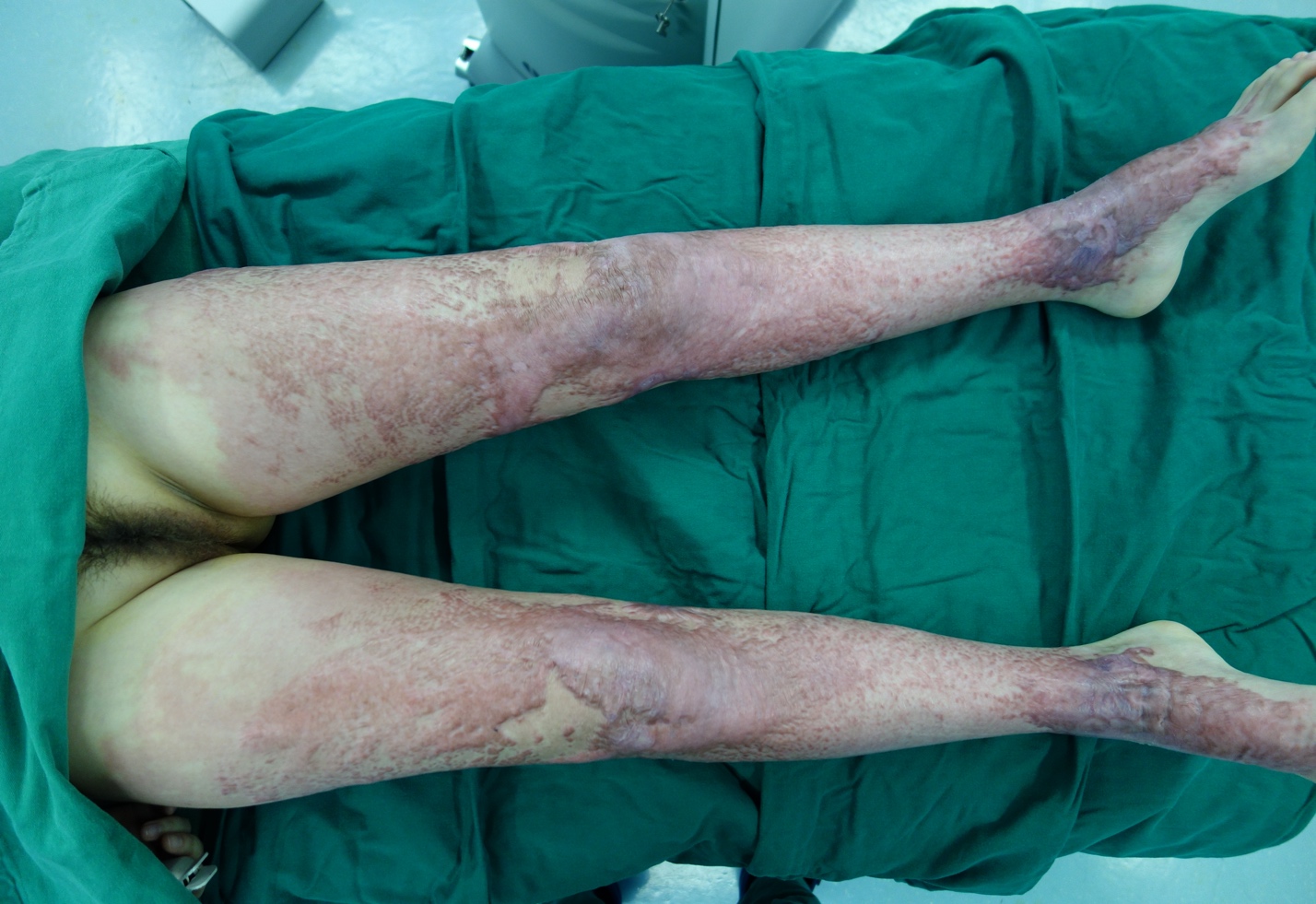


Hypertrophic Scar

Scars across joint

Erythema Scar

Sup. 1a. After 4 months of wound healing (3-month follow-up after the first laser session). The erythematous scarring was significantly improved, the main scar type was hypertrophic, and the scarring at the knee and ankle joints was more obvious than the scarring in other areas.


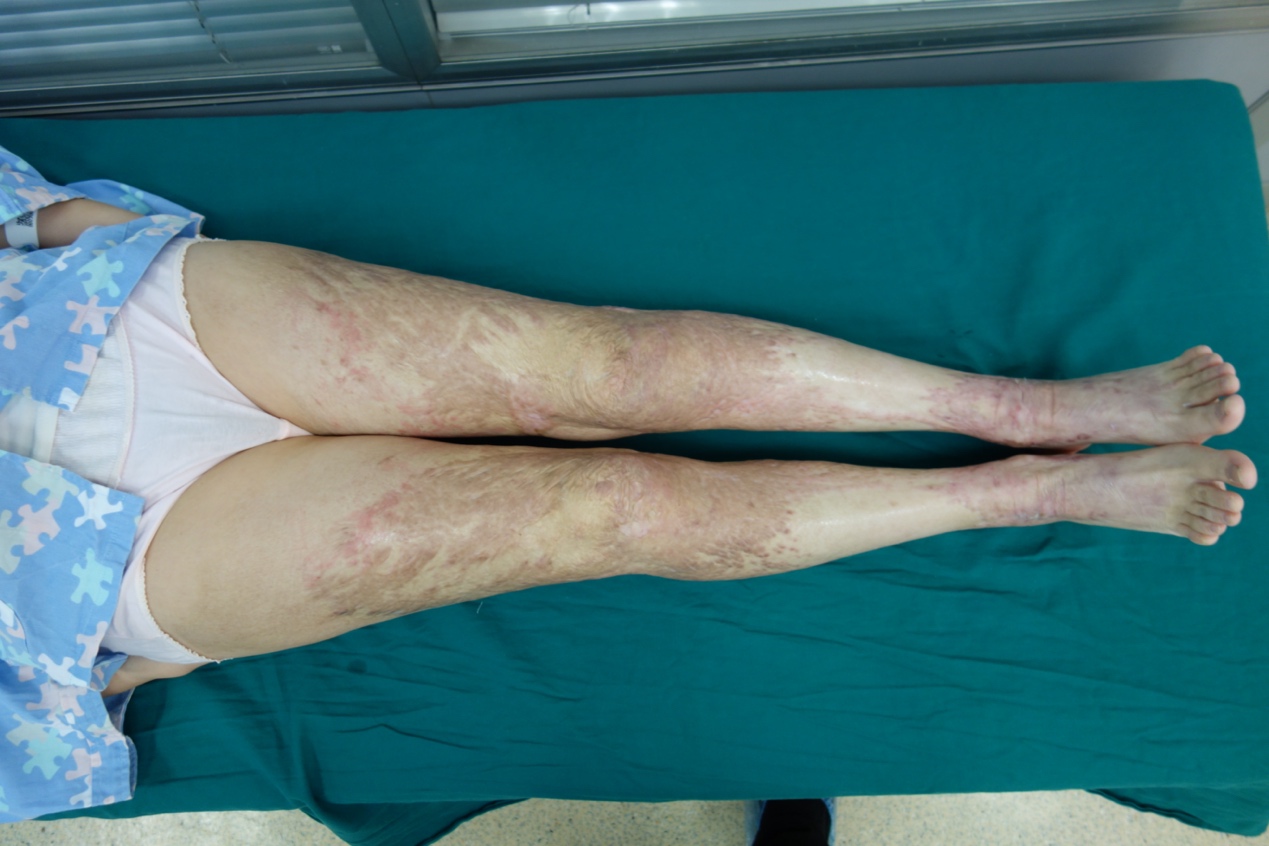


Sup. 1b. Six-month follow-up after 6 sessions of fractional CO_2_ laser treatment. The patient showed no obvious hyperplastic scarring on either lower extremity, the texture of the skin was soft, and the ankle and knee joints showed a normal ROM without functional impairment.


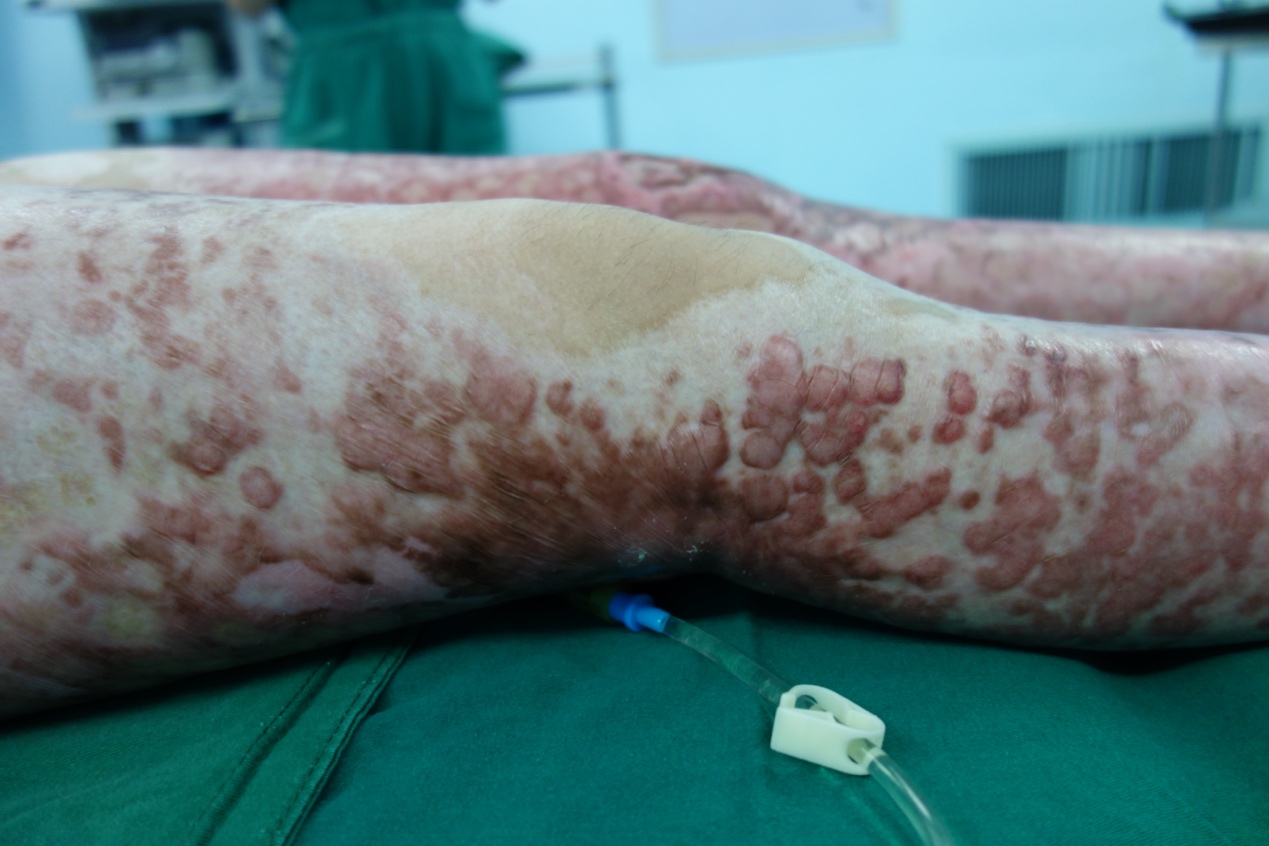


Sup. 2a. Six months after the injury (before the first session). The scar at the knee joint was hard in texture, with obvious stiffness and scar thickening.


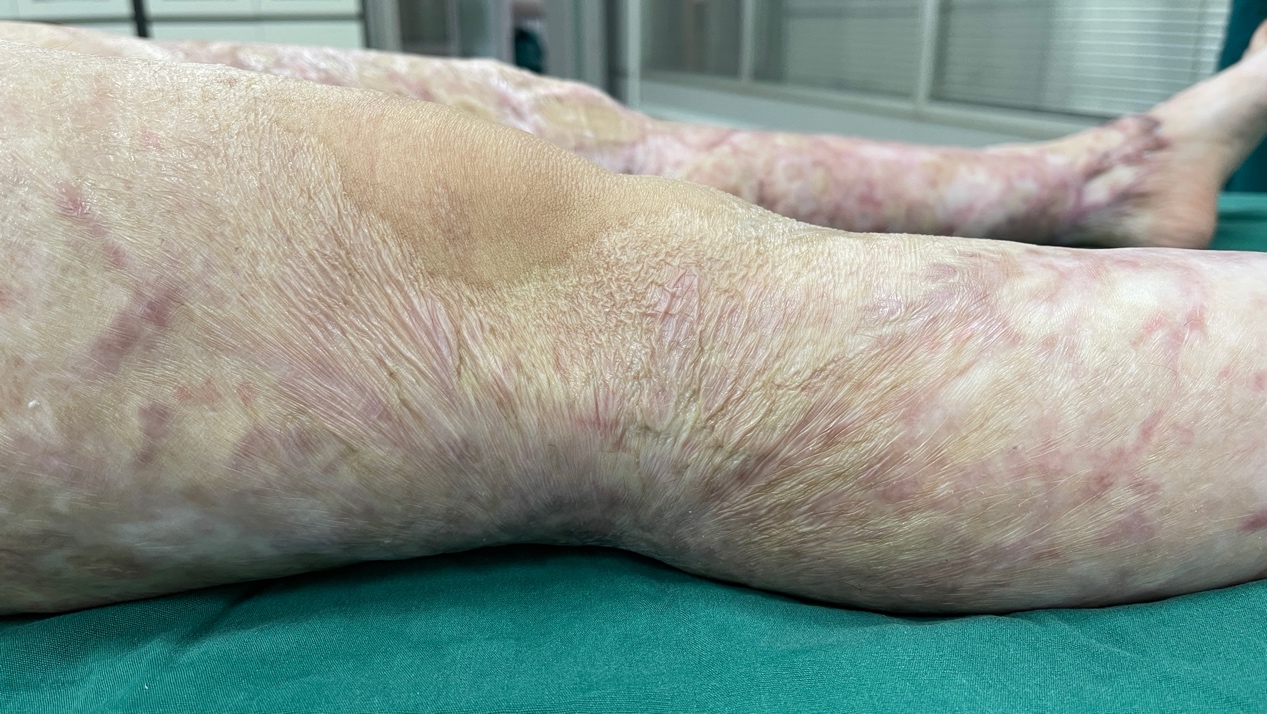


Sup. 2b. Two years after the injury (follow-up after 6 sessions). The ROM was normal, with a soft scar texture and no scar hypertrophy or contracture.
